# Naturally Occurring Culturable Aerobic Gut Flora of Adult *Phlebotomus papatasi*, Vector of *Leishmania major* in the Old World

**DOI:** 10.1371/journal.pone.0035748

**Published:** 2012-05-22

**Authors:** Jaba Mukhopadhyay, Henk R. Braig, Edgar D. Rowton, Kashinath Ghosh

**Affiliations:** 1 Division of Entomology, Walter Reed Army Institute of Research, Silver Spring, Maryland, United States of America; 2 Division of Experimental Therapeutics, Walter Reed Army Institute of Research, Silver Spring, Maryland, United States of America; 3 School of Biological Sciences, Bangor University, Bangor, Wales, United Kingdom; Charité - Campus Benjamin Franklin, Germany

## Abstract

**Background:**

Cutaneous leishmaniasis is a neglected, vector-borne parasitic disease and is responsible for persistent, often disfiguring lesions and other associated complications. Leishmania, causing zoonotic cutaneous leishmaniasis (ZCL) in the Old World are mainly transmitted by the predominant sand fly vector, *Phlebotomus papatasi*. To date, there is no efficient control measure or vaccine available for this widespread insect-borne infectious disease.

**Methodology/Principal Findings:**

A survey was carried out to study the abundance of different natural gut flora in *P. papatasi*, with the long-term goal of generating a paratransgenic sand fly that can potentially block the development of *Leishmania* in the sand fly gut, thereby preventing transmission of leishmania in endemic disease foci. Sand flies, in particular, *P. papatasi* were captured from different habitats of various parts of the world. Gut microbes were cultured and identified using 16S ribosomal DNA analysis and a phylogenetic tree was constructed. We found variation in the species and abundance of gut flora in flies collected from different habitats. However, a few Gram-positive, nonpathogenic bacteria including *Bacillus flexus* and *B. pumilus* were common in most of the sites examined.

**Conclusion/Significance:**

Our results indicate that there is a wide range of variation of aerobic gut flora inhabiting sand fly guts, which possibly reflect the ecological condition of the habitat where the fly breeds. Also, some species of bacteria (*B. pumilus*, and *B. flexus*) were found from most of the habitats. Important from an applied perspective of dissemination, our results support a link between oviposition induction and adult gut flora.

## Introduction

Phlebotomine sand flies (Diptera: Psychodidae) are important vectors of leishmaniasis, Carrión's disease or bartonellosis, and a variety of arboviral diseases [1], [2], [3]. Not only are novel viruses currently being discovered in sand flies, but also different reservoirs are being identified for pathogens and parasites of human diseases, transmitted by sand flies. The distribution areas of sand flies and the diseases they transmit are also expanding. New viral diseases of humans transmitted by sand flies are being reported as well [4], [5], [6], [7]. From a public health viewpoint, however, their greatest importance is as vectors of leishmania [8], [9], [10]. The genus *Phlebotomus* in the Old World, and *Lutzomyia* in the New World, include most of the important vectors of human leishmaniasis. This disease complex is widely distributed in tropical and subtropical regions of the Americas, Africa, southern Europe, and central Asia. It is estimated that some 350 million people in the world are at risk of acquiring leishmaniasis and that approximately 12 million people are currently infected [11], [12]. After malaria, leishmaniasis is the second most important vector-borne parasitic disease and a leading cause of death. There are 500,000 annual new cases of visceral leishmaniasis (VL) or kala-azar in the world and about one-half of them are in India. Bihar, the most affected state in India witnesses almost 90% of the new cases of VL each year with a 10% mortality rate [13], [14].

Cutaneous leishmaniasis is more prevalent throughout the world and causes disfiguration and other associated complications. Cutaneous leishmaniasis and Zoonotic cutaneous leishmaniasis (ZCL), caused by *L. tropica* and *L. major*, respectively, are widely distributed in Turkey, Egypt, Israel, Saudi Arabia and the northern part of India, where mainly *P. sergenti* and *P. papatasi* have been incriminated as the vectors [15]. The Afro-Asian vector of ZCL, *P. papatasi* is widely distributed and is the type species of the genus. The distribution of *P. papatasi* coincides with the distribution of ZCL in most parts of the old world and shows little population differentiation between peridomestic sites and borrows of wild rodents [16], [17]. Despite the demonstrated public health importance, relatively little attempt has been undertaken to block the transmission of this disease by this insect vector.

Information on breeding sites of *P. papatasi* is available from several countries [18], [19]. In India, immature stages of *P. papatasi* have been consistently recovered from cattle sheds and human dwellings in urban areas [20], [21]. In rural areas, they have been found in various habitats: unused poultry houses made of bricks and clay, manure heaps, caves, embankments, dried-up cesspits and latrines [22]. In Egypt, breeding sites of *P. papatasi* have been found in a similar range of ecotopes [23]. Rabbit holes in peri-domestic areas serve as breeding sites, which reduce the indoor abundance of *P. papatasi* in Tunisia [24]. In the Central Asian Republics of the former Soviet Union and neighboring states, burrows of the desert gerbil (*Rhombomys opimus*) are recognized as breeding sites [16]. Caves and dense vegetation of valleys are important in the Judean desert [25]. Dog shelters are important as breeding sites in peri-urban areas of southern Italy [17]. The ease with which *P. papatasi* adapt to an urban environment can be illustrated with the collection of sand flies near the bed and in the bathroom on the second floor in a house in a big city and in another highly urbanized area in southern Italy [26].

Currently, insecticide application at breeding sites is the method of choice for the vector control vis-à-vis control of disease. This control effort targets adult sand flies to bring down populations in order to reduce transmission. Application of insecticide may be limited due to adverse effects on the environment, human health, and the emergence of insecticide resistance in sand flies [27], [28].

Sand flies spend a major part of their life as eggs, larvae and pupae in soil. During the immature stages, they are exposed to a variety of different soil microbes that are available for ingestion. In fact, gravid *P. papatasi* choose oviposition sites by presence of frass and certain soil bacteria [29], [30]. Consequently, it is expected that the sand fly gut harbors a variety of microbial flora. The information on the distribution of the gut flora in feral sand fly populations, especially *P. papatasi*, across different habitat is still lacking. There are a few reports available on other species: from colonized *P. duboscqi* [31], [32], from natural population of *L. longipalpis* [33], and from feral population of *P. argentipes* [34]. A very preliminary study on PCR fingerprinting of the gut flora from Moroccan *P. papatasi* flies identified just two bacteria [32], [35]. There is also a small report on the distribution of gut flora from *P. papatasi* collected in Egypt [32], [35]. Adler and Theodor suggested as early as 1929 that the presence of microbes in the midgut might interfere with *Leishmania* infection [36]. Later, Schlein et al. saw a reduction of infection rate of *L. major* in *P. papatasi* under the influence of yeasts and bacteria [37]. There is little doubt that the developing *Leishmania* in a sand fly gut is exposed to gut flora [38]. In an attempt to develop a strategy to block the transmission of leishmania, which has been demonstrated for some other vector-borne disease pathogens [39], we searched for nonpathogenic gut flora that could be genetically manipulated to release an anti-leishmanial substance and then be reintroduced into the sand fly gut through larval breeding habitats. The long-term objective would be to block or partially disrupt the metacyclogenesis of *Leishmania* sp. by the released product of the recombinant bacterium and thereby render the sand fly incapable of transmitting the disease. This will help to prevent further epidemic outbreak of leishmaniasis. A similar approach has been successfully applied in the development of paratransgenic *Rhodnius prolixus*, a vector of Chagas disease in Central America, with the help of genetically transformed *Rhodococcus rhodnii* [40]. A paratransgenic strategy has also been applied to *Glossina morsitans*, the vector of African sleeping sickness [41], [42]. Additionally, a viral paratransgenic approach has been used to generate a transgenic *Anopheles gambie*, a vector of malaria [43]. A paratransgenic control strategy has also been applied to the glossy-winged sharpshooter with the help of genetically marked *Alcaligenes* sp. [44], [45]. The use of paratransgenesis is explored in the brine shrimp Artemia as a model for controlling infectious diseases in mariculture and in an increasing number of insect groups such as fleas and termites [46], [47], [48]. In mosquitoes, symbiotic yeasts are discussed for control purposes [49]. This is only a short step to consider other eukaryotic symbionts of arthropods [50], [51], [52].

Here we examine the presence and distribution of different aerobic gut microbes of *P. papatasi*, the major vector of ZCL, in different habitats of various geographical parts of the world.

## Materials and Methods

### Collection of field samples

A large number of live sand flies were collected from India, Turkey and Tunisia. Sand flies were captured mainly from human dwellings, sheep sheds, chick pens, rabbit holes and mixed dwellings using light traps, or an aspirator and a flash light. Oral informed consent was obtained from head of households for indoor aspiration of sand flies and/or property owners for shed and outdoor collections that may have included light traps operated overnight. An explanation, in the local language, of the purposes for the collection, how the specimens would be used, the collection methods and any effects the collecting might have on the residents and/or their animals was provided before consent was obtained. Consents were listed in a written log kept by the collectors. Collected sand flies were released in containers with a plaster of Paris bottom. The containers were placed in individual plastic bags with moist cotton to provide necessary humidity for transportation to the laboratory.

### Laboratory colony (control)

We used a laboratory colony of *P. papatasi* originated from field-collected samples from North Sinai, Egypt (PPNS). The colony is maintained at WRAIR following the method of Modi and Rowton [53].

### Preparation of the media

Both liquid and solid agar based sterile media were prepared for the gut bacterial culture. Brain Heart Infusion (BHI) agar plates were prepared following the manufacturer's protocol (BD Biosciences; Cat. # 241830) and liquid culture broth was prepared using Terrific Broth Base (Invitrogen, Cat. # 22711-022).

### Isolation and preservation of bacterial flora

Field collected **s**and flies were identified following the description by Lewis [54]. Only female *P. papatasi* was selected for the isolation of gut flora. In a sterile hood, each sand fly was rinsed in 70% ethanol for two minutes, followed by three quick rinses of sterile 1× PBS. Then the fly gut was dissected out and homogenized in about 60 µl of sterile 1× PBS in a sterile microfuge tube. Forty micro liter of the fly sample homogenate was quickly plated on BHI-agar plate, previously labeled with sand fly origin and number. The plates were subsequently placed in a 33±1°C incubator overnight. The remainder of the homogenate was cryopreserved in a −70°C freezer.

### Selection and culture of clones

After overnight incubation, two to six colonies (depending on the number of colonies obtained from each fly) were picked up using a sterile toothpick and two copies of each colony were cultured in liquid media. The bacterial cultures were allowed to grow overnight in a shaker at 250 rpm at 33±1°C. One culture was used for isolation of DNA while the other was cryopreserved (using 17% sterile glycerol) in a freezer at −70°C. A relatively high incubation temperature was selected for the isolation of the flora because we are mostly interested in generating a recombinant bacterial flora that can grow well and withstand a higher temperature when spread in natural breeding places in a tropical climate.

### DNA extraction, PCR amplification and identification of the bacteria

Genomic DNA was isolated from individual cultures, using DNeasy Blood & Tissue Kit (Qiagen, Cat. #69581). Two sets of primers were used for amplification of the 16S rDNA: a) 533F- 5′-GT TGC CAG CAG CCG CGG TAA-3′ and 1541R- 5′-AAG GAG GTG WTC CAR CC-3′ [55], [56]; and b) 8F-I 5′-AGA GTT TGA TYM TGG CTC AI-3′and 907R-I 5′-CCG TCA ATT CMT TTG AGT TI-3′ [57]. PCR reactions were carried out in a 25 µl reaction mixture containing 25–50 ng of template DNA, 1× PCR buffer (with 2.5 mM MgCl2, 0.2–1 µM of each primer, 0.2 mM dNTPs) and 1 Unit of Taq DNA polymerase (Promega, Cat. #M186). The PCR machine was programmed for the following amplification protocol: one cycle at 95°C for four min; 35 cycles for: 95°C (60 sec), 52°C (60 sec) and 72°C (90 sec) and the final extension step of one cycle at 72°C for six minutes. One non-template control was used for each run. PCR products were detected by agarose gel electrophoresis and purified with QIAquick gel extraction kit (Qiagen, Cat#28704). Nucleotide sequence for each amplicon was determined by using BigDye Terminator v3.1Cycle Sequencing Kit (Applied Biosystems, Cat# 4337455), and 1 U of one of the primers used during PCR amplification. Sequences were blasted and compared with the available sequences at the GenBank database. Isolates were recognized as the same species when their 16S rDNA sequences shared ≥97% homology with complete 16S rDNA.

**Data collection:** After identification, the results were tabulated to show the relative abundance of different species of bacteria isolated from sand flies, collected from different locations and habitats.

### Phylogenetic analysis

The sequences were manipulated in programs SeaView version 4 and MEGA version 5.05 [58], [59]. Alignment of the sequences was based on the secondary structure of their RNAs with the alignment program SINA in the ARB software package using the silva comprehensive ribosomal RNA database version 108 [60]. The alignment was checked by hand. The best evolutionary model among 88 options for the analysis of the alignment was chosen with the help of the program jModeltest version 2 [61], [62]: GTR+I+Γ. Bayesian analysis was performed with the program Mr Bayes version 3.2 [63]. The analysis was carried out with two independent runs with four chains each for 1,000,000 generations of which the first 25% were dismissed. An average standard deviation of split frequencies of 0.0075 was reached, at which point the maximum Potential Scale Reduction Factor for parameter values was 1.002 suggesting conversion. The harmonic mean of the log likelihood of the resulting trees was – 9,612.0. The tree was drawn with the help of the program Figtree version 1.3.1.

## Results

**Figure 1:**
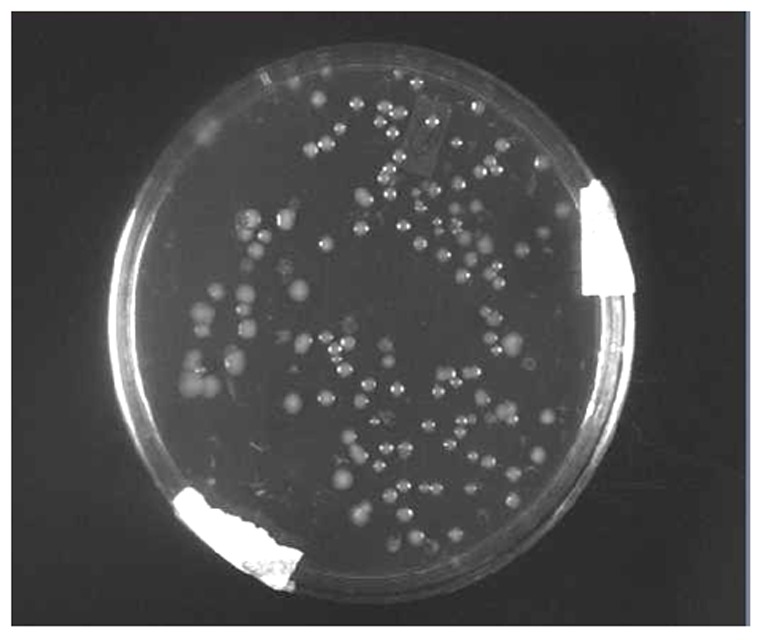
Bacterial clones of sand fly gut flora grown in BHI agar plate showing more than 100 colonies from a single *P. papatasi* female gut.

A total of 107 *P. papatasi* field samples were dissected, of which 43 were collected from Tunisia, 31 originated from Turkey and 33 from India. Of the samples collected, 103 guts were cultured (two guts did not produce any colonies and two others were contaminated during preparation, Table 1). Forty-three female *P. papatasi* from one of our laboratory colonies originating from Egypt (PPNS) were used as control. The number of colonies generated from each fly gut varied widely. From some flies, there were as few as three colonies, while in others there were as many as 153 colonies (Figure 1). Two to six colonies from each sand fly gut were selected for further processing and identification of the flora.

**Table 1:** Description of *P.papatasi* samples collected and screened for gut flora.

Site of collection	Habitat	No. of female flies examined	No of colonies produced	No. of clones examined
Tunisia, SS	Sheep shed	22	514	74
Tunisia, RH	Rabbit hole	21@	447	72
Turkey, SS	Chick/sheep shed - mixed	31#	527	80
Patna, India	Human dwellings	33#,@	518	86
Egypt	Lab colony	43	559	103

# Fly, which did not produce any colony.

@Contaminated sample.

**Figure 2:**
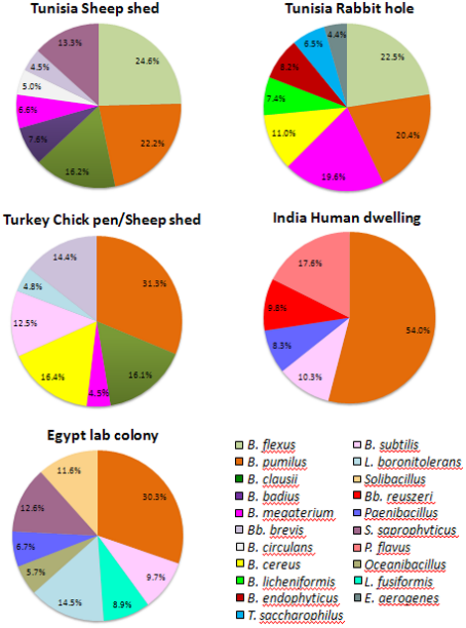
Distribution of gut flora of adult *P. papatasi* females.

The diversity of flora among *P. papatasi* populations, collected from several habitats in three different countries and a laboratory colony is shown in Figure 2. It is evident that there is more variation of the gut flora in the flies collected from animal dwellings of Tunisia and Turkey than in the samples captured from human dwellings in India.

**Figure 3:**
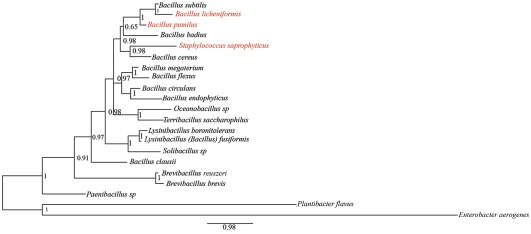
Bayesian 16S tree of gut flora of adult *P. papatasi* females. Posterior probabilities are given along internodes. The scale bar denotes substitutions per nucleotide for the branch lengths. Species that have been implicated in inducing oviposition behavior are highlighted in red.

In *P. papatasi* samples from Tunisia, *Bacillus flexus* was the most dominant bacterium irrespective of the collection habitat. Two other bacteria, *B. pumilus* and *B. megaterium* were also quite common. The flora from *P. papatasi* samples collected in Turkey was diversified with a clear dominance (31%) of *B. pumilus*. Other bacteria, including *B. clausii, B. cereus, B. subtilis* and *Brevibacillus brevis* were also present but at lower frequencies. Aerobic gut microbes in female *P. papatasi* collected from human dwellings of Patna, India, showed less diversity compared to the other two sites; the majority of them (54%) were *B. pumilus*. Four other species were also present in the captured samples but with much lower frequencies (Figure 2). The colonized sand flies from Egypt also showed a relative abundance of *B. pumilus* (30%) with few other microbes. With the exception of two species, *Enterobacter aerogenes* (Enterobacteriaceae, Proteobacteria) and *Plantibacter flavus* (Microbacteriaceae, Actinobacteria), all other bacteria belong to the families Bacillaceae and Paenibacillaceae (Figure 3).

## Discussion

The present study is the most comprehensive evaluation of the distribution of intestinal flora of *P. papatasi* to date, as it describes the abundance of the bacterial gut flora from different habitats of three different countries. Our results show that *P. papatasi* harbor a wide selection of gut bacteria. Roughly, half of the detected bacteria are described for the first time from sand flies and some are described for the first time from insects (Table 2). The diversity of microbes from different habitats strongly suggests that the sand fly gut–microbial association is dependent on microbes in the environment in which those sand flies breed and live.

**Table 2:** Distribution of *P. papatasi* gut bacteria among other hosts.

Bacterial species	other sand fly hosts	other host insects or mites	notes
**Firmicutes**			
Bacillaceae			
*Bacillus flexus* §		*Macrotermes carbonarius* [64]	plants [65], seaweed [66]
*Bacillus pumilus* §	*P. argentipes* [34]	*Apis mellifera* [67]	human and aquaculture probiotic [68], [69], entomopathogen [70], strong oviposition inducer for gravid *P. papatasi* [29]
*Bacillus clausii* §			human probiotic [68]
*Bacillus badius* §			soil [71]
*Bacillus megaterium* §	*P. argentipes* [34]	*Macrotermes carbonarius* [64]	aquaculture probiotic [68], entomopathogen [72]
*Bacillus cereus* §	*P. argentipes* [34]	*Apis mellifera* [67]	human and veterinary probiotic [68], symbiont [73], entomopathogen [74], food pathogen [75], oviposition inducer for gravid *P. papatasi* [29]
*Bacillus licheniformis* §		*Dalbulus maidis* [76]	human, veterinary and aquaculture probiotic [68], very strong oviposition inducer for gravid *P. papatasi* [29]
*Bacillus endophyticus* §			plants [77]
*Bacillus subtilis* §	*P. argentipes* [34]	*Dalbulus maidis* [76]	human and veterinary probiotic [68]
*Bacillus circulans* §			entomopathogen [78]
*Bacillus [Lysinibacillus] fusiformis* §		*Apis mellifera* [79]	bioremediation [80], [81]
*Lysinibacillus boronitolerans* §			soil [82]
*Oceanobacillus* sp.§		*Chironomus* sp. [83]	fermented food [84]
*Terribacillus saccharophilus* §			soil [85]
Paenibacillaceae			
*Brevibacillus brevis* §		*Malacosoma neustria* larvae [86]	plant antifungal [87]; entomo- and human pathogen, *B. laterosporus*: human probiotic [78]
*Brevibacillus reuszeri* §			soil, rhizobacterium [88]
*Paenibacillus* sp§		*Apis mellifera* [89]	entomopathogens [90]
Staphylococcaceae			
*Staphylococcus saprophyticus* §	*P. argentipes* [34]	*Musca domestica* [91]	very strong oviposition inducer for gravid *P. papatasi* [29]
unassigned family			
*Solibacillus* sp.§			forest soil [92]
**Proteobacteria**			
Enterobacteriaceae			
*Enterobacter aerogenes* §	*P. argentipes* [34], *L. longipalpis* [33]	*Apis mellifera* [93]	scale insect symbiont [94], human pathogen [95]
*Enterobacter cloacae* [35]	*P. argentipes* [34], *L. longipalpis* [33]		
*Cronobacter (Enterobacter) sakazakii* [35]		*Stomoxys calcitans* [96], [97]	human pathogen [98]
*Erwinia* spp. [35]		Hemiptera [99]	phytopathogen [100]
*Serratia marcescens* [35]	*L. longipalpis* [33]	*Longitarsus* spp. [101]	entomo- and human pathogen [102], [103]
Moraxellaceae			
*Acinetobacter* sp. [35]	*P. argentipes* [34], *L. longipalpis* [33]	*Bactericera cockerelli* [103]	human pathogen [104]
Pseudomonadaceae			
*Pseudomonas aeruginosa* [35]	*L. longipalpis* [33]	*Musca domestica* [105]	human pathogen [106]
*Pseudomonas* spp. [35]	*P. argentipes* [34]		entomo-, phyto- and human pathogen [106]
**Actinobacteria**			
Microbacteriaceae			
*Plantibacter flavus* §			grass [107]
*Microbacterium* spp. [32]	*P. argentipes* [34], *P. duboscqi* [32]	*Bemisia tabaci* [108]	human pathogens [109]
Propionibacteriaceae			
*Propionibacterium* sp. [35]		*Psoroptes ovis* [110]	human and veterinary probiotic [111], [112], *P. acne*: human pathogen [113]
**Chloroflexi**			
Chlorobacteria spp.¶ [32]	*P. duboscqi* ¶ [32]		filamentous green non-sulfur bacteria [114]

§ this report;

¶ immature stages only.

The gut flora in sand flies collected from sheep sheds and rabbit holes in Tunisia and Turkey showed more diversity than other groups. However, no significant differences in the distribution of the microbes were observed in the gut of sand flies collected from sheep shed or rabbit holes in Tunisia. Among the predominant flora observed from the flies collected from these two habitats, *B. cereus* is a potential human pathogenic bacterium [115]. The same is true for *En. aerogenes*, which has also been found to cause infections [116]. An interesting case is *B.circulans* because in the older Russian literature, it was mentioned together with *B. mycoides* as an entomopathogen of the gut of larval fleas [117]. Later, it was also recognized as a gut pathogen of mosquito larvae [118]. More recently, *B. circulans* was investigated as a potential probiotic for juvenile rohu in freshwater fish aquaculture [119]. Among the bacterial flora, *B. megaterium* and *B. flexus* are reported not only as non-pathogenic but also having some beneficial effect as probiotics [68], [69].

The sand flies in Tunisia and Turkey were collected from animal shelters including sheep sheds, rabbit holes and poultry pens. Usually, the soil in and around these areas is contaminated by the excreta of the animals and other environmental contaminants making the soil a fertile medium for the growth of coprophilic bacteria. This contamination could explain the diversity of the bacterial flora found in the sand fly gut collected from these habitats. The diversity may be accentuated in places where animal shelters are in close proximity to agricultural land where the use of biofertilizers add more microbes to the nearby animal shelters. One example of this is the presence of *B. megaterium*, which have been found to have a good growth enhancement effect and yield, and have been used as a biofertilizer [120], [121].

The diversity of bacterial population is somewhat restricted in the sand flies captured from India. Although other bacteria are present in less frequency, *B. pumilus* is the predominant bacterium found in the sand flies from Patna, India. Here, the majority of *P. papatasi* were obtained from human dwellings which is consistent with the anthropophilic nature of *P. papatasi* [122], [123]. Blood-fed sand flies use the loose soil in the dark corners inside the mud houses as the most favorable place for oviposition [124]. Larvae are only exposed to the microbes inside the mud-house but not to the excreta of animals and other environmental contaminants. This may explain the lower diversity of the gut flora isolated from sand flies captured from human dwellings.

An unexpected result is that the Egypt lab colony seems to show a higher or similar diversity of bacterial flora as samples originated from any of the natural habitats. This observation might be explained by the fact that the sand fly larvae are maintained in the laboratory on a diet composed of rabbit chow and rabbit feces, which are additional resources of gut flora and might have contributed to the bacterial diversity. Blood-fed females defecate gut bacteria along with the remains of the blood meal. Sand fly larvae are coprophagous. Therefore some gut bacteria are vertically transmitted to the next generation.

*Bacillus pumilus*, one of the most dominant bacteria of all the populations, is a Gram-positive, aerobic, rod-shaped, soil-dwelling bacterium. Like other *Bacillus* species, the spores produced by *B. pumilus* are more resistant than vegetative cells to heat, desiccation, UV radiation, γ-radiation, H_2_O_2_, and starvation. This species has been found in extreme environments such as the interior of Sonoran desert basalt and the Mars Odyssey spacecraft [125], [126]. The presence of *B. pumilus* in higher numbers in sand flies collected from human dwellings might be significant from the microbiological point of view as it has been shown that *B. pumilus* exhibits strong antifungal and antiviral activity [127], [128]. Schlein et al. postulated that some gut bacteria might help to destroy fungi, thereby indirectly helping the development of *Leishmania* in the sand fly gut [37]. It is not clear at this point if *B. pumilus* is engaged in antifungal activity in the sand fly gut at all or if it acts together with other closely related *Bacillus* species or in combination with other gut factors to make the sand flies mycosis free. A fungi-free gut may help *Leishmania* survive which would make sand flies a more competent vector.

In the present study, a large number of *Bacillus* species was identified from *P. papatasi*. A preliminary study reported a different profile of bacteria. Species of *Enterobacter* and *Cronobacter* were isolated in greatest abundance from *P. papatasi* from Egypt by Dillon and others [35]. The authors emphasized that they used a rather selective medium and culture conditions. However, in a previous study on *P. argentipes* from India, we found a higher abundance of *Enterobacteriaceae* [34].

For the New World sand flies, Oliveira et al. found a high percentage of *Staphylococcus* sp. (28%) and *B. thuringiensis* (18%) in *Lutz. longipalpis* samples collected from Lapinha cave, Brazil [129]. They also recorded a relatively low percentage of *En. cloacae* (9%). We believe that these variations in the abundance of different bacteria from feral populations of sand flies are due to the ecological setting of their breeding habitat and species related.

The phylogenetic analysis shows strong support for all clades with the exception of *B. badius*. It is very reassuring that with the exception of *B. megaterium*, all species of our phylogenetic analysis using 16SRNA only showed similar relationship to a recent whole-genome phylogenetic analysis of the family Bacillaceae [130]. *Staphylococcus* species often clusters in 16S phylogenies within clades of *Bacillus* species [131]. *Bacillus fusiformis* of the literature cited here should be recognized as a *Lysinibacillus* species [119]. Species that have been observed by Radjame et al. to induce oviposition behavior in gravid *P. papatasi* females do not form a strict clade but cluster in a bigger group among the species recovered in this study [29].

Since there is very little information on the symbiotic association of bacteria with sand flies, gut colonization of bacteria is believed to be dependent on the larval food and the breeding soil. The larvae acquire many soil microbes during their immature stages of development which are believed to survive during the transformation until the adult emergence as reported in *P.duboscqi* by Volf et al. [31] (unpublished observation, Ghosh) However, in nature, adult sand flies may also have the opportunity to ingest microorganism through contaminated sugar meal derived from leaves, fruits or aphid honeydew taken between blood meals. Some sand fly species, in particular *P. papatasi*, may ingest microorganism from the plant cuticle while sucking the plant juice [132]. This explains some of the plant-associated bacteria found in our study.

Radjame et al. found that several soil bacteria significantly enhance the oviposition response of *P. papatasi* females [29]. The most pronounced effect was observed with *B. firmus* (P 0.00001 in cattle sheds), followed by *S. saprophyticus* (0.0003 in termite mounts and 0.002 in human dwellings), and *B. licheniformis* (0.0007 in cattle sheds, 0.003 in termite mounts and 0.0091 in human dwellings). Importantly, *B. pumilus* also induced oviposition of sand flies in cattle sheds significantly [29]. More studies are needed to find out the ability of *Bacillus* species to induce oviposition behavior under various conditions, especially in human dwellings. Of all the species considered, *B. pumilus* is particularly attractive because it has been recovered from all our study sites.

This study succeeded in identifying several candidate species for paratransgenesis in *P. papatasi*: *B. flexus*, *B. pumilus*, *B. licheniformis*, *B. megaterium* and *B. subtilis*. These bacteria are genetically tractable and trackable and are often used as probiotics. Most importantly, *B. pumilus* and *B. licheniformis* have been proposed as strong oviposition inducers for gravid *P. papatasi* [29]. The latter fact identifies those bacteria as true symbionts and not merely as environmental contaminants, which might be crucial for the dissemination of the bacteria into sand fly populations.
